# Supplementary material for: Information feedback in relative grading: Evidence from a field experiment
Source: PLoS One. 2020 Apr 20;15(4):e0231548. doi: 10.1371/journal.pone.0231548 (PMC7170246; doi:10.1371/journal.pone.0231548)
Supplement: S1 File — The instruction to replicate the tables and figures in “Information Feedback in Relative Grading: Evidence from a Field Experiment” by Shinya Kajitani, Keiichi Morimoto and Shiba Suzuki. (PDF) [file pone.0231548.s001.pdf]

Steps to replicate the tables and figures in “Information Feedback in Relative Grading: Evidence from a Field Experiment” by Shinya Kajitani, Keiichi Morimoto and Shiba Suzuki

Instructions for replication of the results are included below. The program is in Stata (*.do) languages. This was run in Stata/MP 15.1.

1. There are two files: Stata program file (‘S3_File.do’) and a CSV file (‘S2_Dataset.csv’). These need to be copied and placed at your computer in the same directory.

2. Install the following ado-files for Stata before running the program.
   - vioplot
   - fitstat

3. Run ‘S3_File.do’ to perform statistical analyses for all figures and tables in the main text.
