# Supplementary material for: Information feedback in relative grading: Evidence from a field experiment
Source: PLoS One. 2020 Apr 20;15(4):e0231548. doi: 10.1371/journal.pone.0231548 (PMC7170246; doi:10.1371/journal.pone.0231548)
Supplement: Attachment — Submitted filename: PLOS_One_Review.docx [file pone.0231548.s004.docx]

**“Information feedback in relative grading: Evidence from a field experiment”**

PLOS-One

In this paper, the authors look at how effort responds to information about relative position in a classroom graded on the curve. The primary treatment arm randomly assigns students to receive information about their midterm exam grade relative to the population of students. Then, the authors measure how this affects performance on the final exam. As a result of this information intervention, higher ability students reduce their motivated relative to lower ability students.

This paper has chosen an important topic and an ambitious approach to studying it. I have no concerns about this being of broad interest.

I have two primary concerns with the paper and a few smaller comments about papers they may want to cite:

By separating the top 40 students, the interpretation of information feedback becomes challenging. Moreover, the treatment assignment weights are different between classroom 1 and classrooms 2-4.

- I’m a bit confused about Table 3, where there are class-level indicator variables. In this case, dropping Class 1 should only change the precision of the estimates of other covariates, but shouldn’t change the estimation of the treatment effects, because there is no within-class-1 variation in treatment. So, I think you could even drop columns (2a) and (2b) and say it’s taken care of by the indicator variables.
- When you think about the treatment on the treated, it is also challenging to think about how this classroom affects that. If I’m not in Class 1, then I already know something about my relative score, so it’s a little strange. I would like to see this explained a bit in the text.

The fact that the courses are not explicitly graded on a curve is a little strange. The curving is more important for the lower performing students because it can only move scores upwards. I would like to see the paper mention how an asymmetric curve may affect lower ability students differently from higher ability ones.

A few papers that I would cite are:

1. Gill, David, et al. "First-place loving and last-place loathing: How rank in the distribution of performance affects effort provision." *Management Science* 65.2 (2019): 494-507.

- This could help inform the discussion of how rankings matter for students.

1. Bedard, Kelly, and Stefanie Fischer. "Does the response to competition depend on perceived ability? Evidence from a classroom experiment." *Journal of Economic Behavior & Organization* 159 (2019): 146-166.
2. "Threshold Incentives and Academic Performance," Pol Campos-Mercade and Erik Wengström
3. Brownback, Andy. "A classroom experiment on effort allocation under relative grading." *Economics of Education Review* 62 (2018): 113-128.

- These three can address the existing literature on how perceptions or realities about relative ranking affect effort choices.
